# Spleen contraction elevates hemoglobin concentration at high altitude during rest and exercise

**DOI:** 10.1007/s00421-020-04471-w

**Published:** 2020-09-10

**Authors:** Erika Schagatay, Alexander Lunde, Simon Nilsson, Oscar Palm, Angelica Lodin-Sundström

**Affiliations:** 1 Department of Health Sciences, Mid Sweden University, 831 25 Östersund, Sweden; 2 Swedish Winter Sports Research Centre, Mid Sweden University, Östersund, Sweden; 3 Department of Nursing Sciences, Mid Sweden University, Sundsvall, Sweden

**Keywords:** High altitude, Hematocrit, Hemoconcentration, Spleen volume, Spleen size

## Abstract

**Purpose:**

Hypoxia and exercise are known to separately trigger spleen contraction, leading to release of stored erythrocytes. We studied spleen volume and hemoglobin concentration (Hb) during rest and exercise at three altitudes.

**Methods:**

Eleven healthy lowlanders did a 5-min modified Harvard step test at 1370, 3700 and 4200 m altitude. Spleen volume was measured via ultrasonic imaging and capillary Hb with Hemocue during rest and after the step test, and arterial oxygen saturation (SaO_2_), heart rate (HR), expiratory CO_2_ (ETCO_2_) and respiratory rate (RR) across the test.

**Results:**

Resting spleen volume was reduced with increasing altitude and further reduced with exercise at all altitudes. Mean (SE) baseline spleen volume at 1370 m was 252 (20) mL and after exercise, it was 199 (15) mL (*P* < 0.01). At 3700 m, baseline spleen volume was 231 (22) mL and after exercise 166 (12) mL (*P* < 0.05). At 4200 m baseline volume was 210 (23) mL and after exercise 172 (20) mL (*P* < 0.05). After 10 min, spleen volume increased to baseline at all altitudes (NS). Baseline Hb increased with altitude from 138.9 (6.1) g/L at 1370 m, to 141.2 (4.1) at 3700 m and 152.4 (4.0) at 4200 m (*P* < 0.01). At all altitudes Hb increased from baseline during exercise to 146.8 (5.7) g/L at 1370 m, 150.4 (3.8) g/L at 3700 m and 157.3 (3.8) g/L at 4200 m (all *P* < 0.05 from baseline). Hb had returned to baseline after 10 min rest at all altitudes (NS). The spleen-derived Hb elevation during exercise was smaller at 4200 m compared to 3700 m (*P* < 0.05). Cardiorespiratory variables were also affected by altitude during both rest and exercise.

**Conclusions:**

The spleen contracts and mobilizes stored red blood cells during rest at high altitude and contracts further during exercise, to increase oxygen delivery to tissues during acute hypoxia. The attenuated Hb response to exercise at the highest altitude is likely due to the greater recruitment of the spleen reserve during rest, and that maximal spleen contraction is reached with exercise.

## Introduction

Spleen contraction with release of stored erythrocytes resulting in elevation of the circulating red blood cell volume occurs in various situations and species, including humans (Stewart and McKenzie 2002). This response has been observed in humans during exercise (Laub et al. 1993), exposure to apnea (Schagatay et al. 2001; Bacovic et al. 2003; Richardson et al. 2005) or a hypoxic environment (Richardson et al. 2008). While hypoxia is known to be an important trigger of spleen contraction (Richardson et al. 2009) it is further enhanced by the hypercapnia developing during apnea (Richardson et al. 2012), resulting in a more powerful contraction during apnea compared to simulated high altitude despite similar levels of hypoxia (Lodin-Sundström and Schagatay 2010). The spleen has sympathetic innervation and contraction is likely resulting from both neural input and catecholamine release (Stewart and McKenzie 2002), and the contraction was shown to be active, rather than due to a passive collapse (Bacovic et al. 2003).

Spleen function as a dynamic red blood cell reservoir is well described during apneic diving, but less studied at high altitude. Spleen contraction was first observed after diving shifts in professional Ama breath-hold divers (Hurford et al. 1990). The progressive elevation of hemoglobin concentration (Hb) resulting from spleen contraction across serial apneas was found to prolong apneic duration in intact subjects while this response was not found in splenectomized subjects (Schagatay et al. 2001). In another study, spleen volume was found to correlate with competitive apneic diving performance in elite freedivers (Schagatay et al. 2012). Spleens were also larger in professional Bajau breath-hold divers, than in a non-diving population (Ilardo et al. 2018), which is functionally logical as the increase in circulating Hb will increase blood gas storage capacity as well as carbon dioxide buffering, both contributing to prolonged apneic duration. The decrease in spleen volume typically increases the total amount of circulating erythrocytes by 3–6% with individuals responding with up to 10% increases (Richardson et al. 2008). This natural blood-boosting was also found to be active during exercise in chronically hypoxic patients with chronic obstructive pulmonary disease (COPD), where the most hypoxic patients were found to have the largest spleens and the most pronounced spleen contractions with exercise (Schagatay et al. 2015).

After maximal spleen contraction, it takes approximately 10 min before the spleen red cell supply is re-stored and the spleen has expanded to the resting volume (Schagatay et al. 2005). The spleen function as a dynamic red cell reservoir will not only transiently increase oxygen storage and delivery during exercise or hypoxic exposure, but lower blood viscosity between bouts of exercise or severe hypoxia and thereby reduce the sheer stress and thereby the work of the cardiovascular system (Schagatay et al. 2015).

As one of the major stimuli initiating spleen contraction is hypoxia, it is evident that spleen contraction could occur and be beneficial also at high altitude, especially during bouts of exercise leading to aggravated hypoxia. A study suggesting that spleen contraction occurs at high altitude was an observation of Hb increase across apneas at high altitude in a group of three climbers, a response that diminished with increasing altitude (Richardson and Schagatay 2007). However, actual spleen contraction at high altitude has up to now not been observed. Aiming to study this possibility, a recent study using handgrip exercise to induce spleen contraction found an effect on low altitude but not at high altitude, and the hematological response resulting from spleen contraction at low altitude was also absent at high altitude (Purdy et al. 2018). This seems to contradict the finding in a longitudinal study that spleen contraction during apnea and exercise was enhanced after a climb to the summit of Mt Everest (Engan et al. 2014), suggesting an important role of the spleen at altitude. It was also recently observed that elite high altitude climbers, going to the summit of Mount Everest at 8848 m, had larger spleens than trekkers to Mount Everest Base Camp at 5300 m (Schagatay et al 2020).

While this hypoxia-induced mechanism would thus likely be beneficial for high altitude performance, and has been demonstrated to occur at simulated high altitude (Richardson et al. 2008), there is incomplete understanding of if, when and to what extent this response is actually present at high altitude. Our aim was, therefore, to reveal if spleen contraction and Hb elevation occur at high altitude, if this response is enhanced during whole-body exercise at high altitude, and whether the response is affected by the altitude.

## Methods

### Participants

Based on power calculations on the studied variables, the aim was to include *N* ≥ 10 participants in the analysis. This suggested that at least 12 participants should be recruited, to allow some loss due to causes such as illness. Twelve healthy lowlanders (six men and six women) were recruited among university students planning a trek to 4200 m altitude in the Nepali Himalayas. They volunteered to participate in this study without benefits or payment, and knowing they could withdraw at any time without consequences. One woman did not complete all tests, and was excluded from the analysis. Mean (SD) anthropometric data for the remaining 11 subjects were age 26 (9) years, height 177 (10) cm and weight 71 (10) kg, and BMI 22.8 (2.1). Vital capacity was 5.1 (1.3) L; 107.5 (10.7)% of predicted; men 6.02 (0.9) L; 107.4 (13.9)% of predicted and women 4.0 (0.3) L; 107.5 (6.6)% of predicted. Men were taller [184 (5) cm] and heavier [79 (5) kg] than the women [167 (5) cm; and 63 (8) kg], respectively (*P* < 0.01), and had larger lungs (*P* < 0.001), but there were no other differences between the sexes.

Participants were given a written and oral description of the procedures and potential risks involved, after which they signed a consent form. They also filled out a medical questionnaire, and all were to their own knowledge healthy. The study protocol had been approved by the Regional Committee for Medical and Health Research Ethics in Umeå, Sweden (Dnr: 2011-454-31M) and the Nepali Health Research Council (NHRC; Ref. No. 1079), and it complied with the 2004 Declaration of Helsinki. Baseline testing at a lowest altitude was done in Kathmandu at 1370 m, as this was where all the participants gathered before starting the trek.

### Procedures

**Fig. 1:**
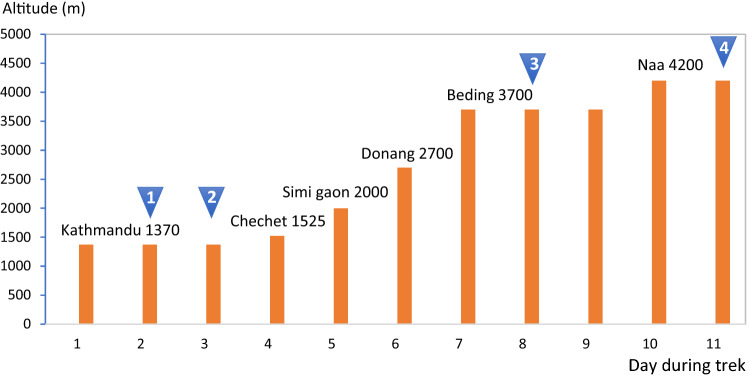
The field measurements were conducted during 10 days at three different locations in the Rolwaling valley in the Nepali Himalayas. All subjects followed the same ascent profile. Altitudes for the locations where participants slept are shown. Arrows mark measurement days; 1: collection of background data and 2: step test in Kathmandu; 3: step test in Beding; 4: step test in Naa

The measurements were conducted in the field during the first part of a longer trek in Rolwaling in the Nepali Himalayas at three different altitudes: Kathmandu 1370 m; Beding 3700 m and Naa, 4200 m above sea level (Fig. 1). During day 2 in Kathmandu, anthropometric data were collected and previous and recent history of altitude was noted. None of the subjects had been above 3000 m during the last 3 months. During the camping trek, each new altitude was reached in the evening, and one night was spent at that altitude after which the trek continued in the morning, except at the altitudes for measurements, where 2–3 days were spent (Fig. 1). The trek involved 5–8 h of trekking per day except testing days, carrying 14–18 kg per participant. The main part of the cargo: equipment necessary for camping, cooking, food and materials for measurements were carried by 45 porters. No participant took any drugs against symptoms of acute mountain sickness (AMS) during the study period and participants were asked to fill out the Lake Louise self-assessment protocol (LLQ) every morning for symptoms of AMS (Roach et al 1993).

The first exercise test was done in Kathmandu on day 3 after arrival and on the other locations, testing was done during the second day at that altitude, after arriving in the evening and sleeping one night (Fig. 1). Testing started in the early morning and was carried out in the same individual order at each location. Measurements of one subject took approximately 30 min. The subjects did not eat or drink for at least 1 h prior to testing. To detect any dehydration, a morning urine sample of 30 ml was collected in a white cup and the color of the urine compared with a color chart graded 1–8, where 1–3 is normal and 4–8 dehydrated. The chart is commonly used and acceptable to estimate dehydration in field conditions (Shirreffs 2000). No one was considered dehydrated during the days of testing.

### Exercise test

A modified Harvard step test was used for the exercise test because it was considered to mimic daily motion patterns and would be easily repeatable for all subjects at all altitudes, is simple to standardize at different locations and because no heavy equipment is needed. The test started with the participant resting for 10 min in an upright position, while measurement probes were applied. Five minutes before the exercise test minute by minute measurements of spleen volume started and heart rate (HR), arterial oxygen saturation (SaO_2_), end tidal alveolar carbon dioxide (ETCO_2_) and respiratory rate (RR) were recorded continuously. Approximately 1 min before the subject started to exercise, a capillary blood sample on the finger was taken for hemoglobin concentration (Hb). Directly after blood sampling, the subject stood up and remained standing for 1 min while listening to a metronome ticking at 1-s interval, the rhythm used when stepping up and down a box with a height of 31 cm. The subject stepped up and down for 5 min and changed starting leg on a signal after 2.5 min. During the exercise, no spleen or blood measurements were done. After the exercise, the subject sat down and immediately a second capillary blood sample was taken and measurements of the spleen started and continued at 1-min intervals for 10 min during recovery. After 10 min of post-exercise rest, a third capillary blood sample was collected.

### Measurements

The spleen measurements during 5 min prior to exercise, and 10 min after the step test was done with a portable ultrasonic imager (M-Turbo ultrasound system, FUJIFILM Sono Site Inc, Bothell, WA, USA), with the probe C60x/5–2 MHz (Transcluser Sono Site Inc, Bothell, WA, USA). Two pictures were taken every minute for determining the three-axial maximal diameters of the spleen for length (*L*), thickness (*T*), and width (*W*). These values were used to calculate the total spleen volume using the equation *L*π (*WT* − *T*^2^)/3, developed by Pilström (Schagatay et al. 2005).

The capillary finger blood samples for Hb, taken 1 min before exercise, directly after exercise, and after 10 min of rest were collected in triplicate in microtainer cuvettes, and analyzed directly in a portable hemoglobin analyzer (Hemocue AB, Ängelholm, Sweden).

Cardiorespiratory variables: HR, SaO_2_, ETCO_2_ and RR were measured continuously across the exercise test using a combined pulse-oximeter and capnograph (Medair Lifesense LS1-9R, Nonin Medical Inc, Medair AB, Hudiksvall, Sweden) and stored in a memory unit (Trendsense, Nonin Medical Inc, Medair AB, Hudiksvall, Sweden). The pulse-oximetry sensor was placed on the subjects’ middle finger and a sampling hose for respiratory variables was placed under the subjects’ nose.

### Analysis

Mean baseline spleen volumes during the 5-min periods preceding the exercise test were compared between altitudes. For spleen contraction during exercise, the first post-exercise volume and the last value of 10 min of rest after the step test were compared to the last value during rest before the exercise. For Hb, individual means of the three samples were used, and pre- and post-exercise and post-rest values were compared. Cardiorespiratory variables compared were mean values collected during the last minute of sitting rest, the last minute of exercise, and the last minute of the 10 min post-exercise rest. Subjects served as their own controls. One subject could not keep the stepping pace with the metronome during the last minute at the highest altitude, but the test was completed and peak values were included. Baseline variables and responses were compared between sexes. There were no differences found in any of the studied variables and, therefore, all subjects were treated as one group. All 11 subjects were included in all measurements, except in RR recordings, where one subject lacked data for the main part of the test, and was excluded from RR—analysis at all altitudes, thus RR values are based on *n* = 10 subjects. The LLQ score from 3 days in the testing locations Beding at 3700 m and 3 days in Naa at 4200 m, including the first days of exposure to the respective altitudes, were used to calculate a mean individual score, which was analyzed for correlation with measured variables.

Statistical analysis was done using ANOVA followed by Student´s paired *t* tests. Baseline values for spleen volume and Hb were compared between altitudes using a univariate one-way repeated measures ANOVA. Similarly, within-location values for exercise-induced spleen volume and Hb changes were compared using one-way repeated measures ANOVA. To assess the interaction between altitude (independent variable) and exercise (independent variable) on spleen contraction (dependent variable) and Hb change (dependent variable), a two-way repeated measures ANOVA was conducted. Simple main effects was assessed using separate one-way repeated measures ANOVAs with post hoc test using Bonferroni adjustment for multiple comparisons to assess for significant differences. Pearson´s correlation analysis was used for correlation analysis. G power (3.0.10) was used to calculate observed power for selected variables. Significant difference was accepted at *P* < 0.05.

## Results

### Spleen volume

**Fig. 2:**
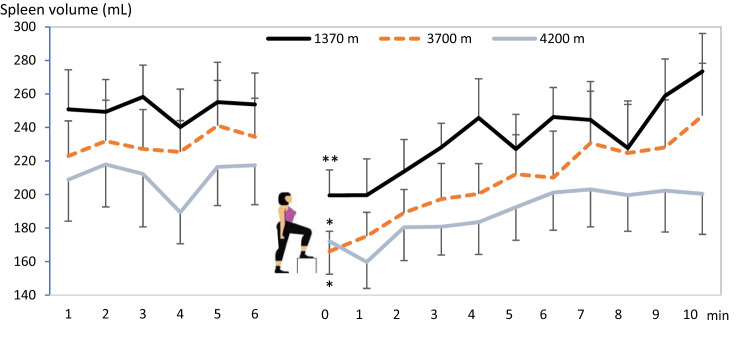
Mean (SE) spleen volume across the exercise test at 3 altitudes for 11 participants. Difference between the last minute value during rest and first value after exercise is indicated by *P < 0.05 and **P < 0.01

**Fig. 3:**
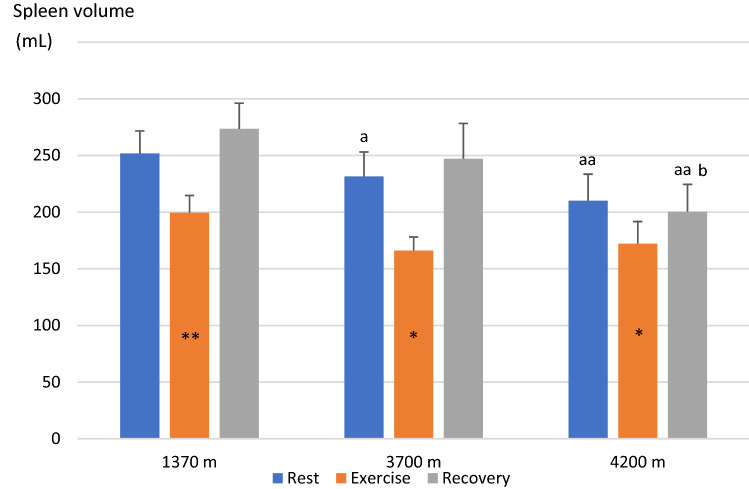
Mean (SE) spleen volumes during 5-min rest before step test, directly after the 5-min step test, and after 10-min rest at 3 altitudes for 11 participants; Kathmandu 1370 m, Beding 3700 m and Naa 4200 m. Significant difference between baseline value and first post-exercise value within altitudes is indicated with **P < 0.01 and *P < 0.05. At 10 min post-exercise, spleen volumes were back to baseline at all altitudes (all NS). Differences between baseline volumes at Kathmandu and other altitudes is indicated with a above columns (a for *P* < 0.05; aa for *P* < 0.01). There was no difference between Beding and Naa for rest and exercise (NS). Post-exercise volumes were different between Beding and Naa (b for *P* < 0.05)

Mean (SE) baseline spleen volume during the last 5-min rest preceding the exercise test was 252 (20) mL at 1370 m, and it was subsequently reduced with increasing altitude, to 231 (22) mL at 3700 m (*P* < 0.05, power = 0.74) and 210 (23) mL at 4200 m (*P* < 0.01, power = 0.53, compared to 1370 m; *P* = 0.124, power 0.12, compared to 3700 m; Figs. 2 and 3). After the step test, spleen volume had decreased at all altitudes, to 199 (15) mL at 1370 m (by 53 mL; 21%), 166 (12) mL at 3700 m (by 65 mL; 28%), and 172 (20) mL at 4200 m (by 38 mL; 18%; all *P* < 0.05, (power = 0.99, 1.00 and 0.99, respectively) compared to baseline; Figs. 2 and 3). Post-exercise volumes did not differ between altitudes (NS, power = 0.71–0.99). Changes in volumes with exercise were 52 (14) mL at 1370 m, 64 (19) mL at 3700 m and 38 (12) mL at 4200 m (*P* = 0.158 from 3700 m, power 0.82; Fig. 5). Neither were there any significant differences between altitudes in the relative changes (NS).

During the 10-min period of rest after exercise, spleen volume gradually increased back to baseline levels at all altitudes (all NS from baseline values; Fig. 2). At 10 min, it was 273 (23) at 1370 m, 247 (31) at 3700 m (*P* = 0.329, power = 0.98 from 1370 m), and 200 (24) mL at 4200 m (*P* < 0.001, power = 0.99 from 1370 m; *P* = 0.097, power 0.99 from 3700 m; Fig. 3).

### Hemoglobin concentration

**Fig. 4:**
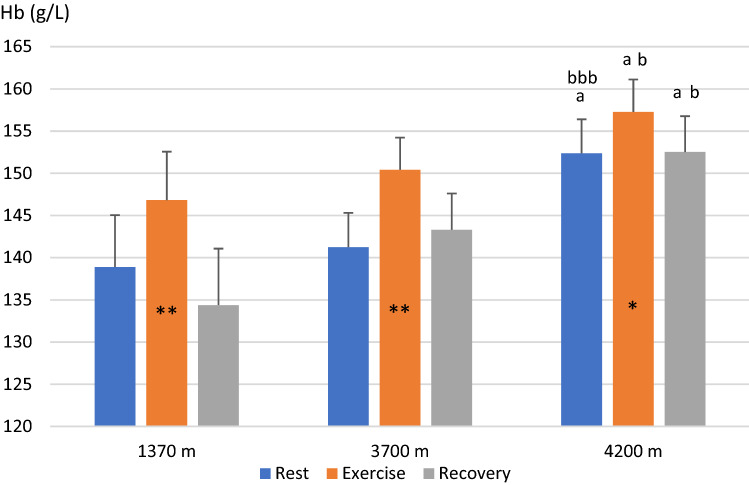
Mean (SE) hemoglobin concentration (Hb) during rest before exercise, during the last minute of the step test, and 10 min after exercise for 11 participants at 3 altitudes; in Kathmandu 1370 m, Beding 3700 m and Naa 4200 m. Significant difference between baseline value and first post-exercise value within altitudes is indicated with **P < 0.01 and *P < 0.05. At 10 min post-exercise, spleen volumes were back to baseline at all altitudes (all NS). Differences between baseline volumes at Kathmandu and other altitudes are indicated with a above columns for *P* < 0.05. There was also a difference between Beding and Naa for exercise and post-exercise volumes (b for P < 0.05; bbb for *P* < 0.001)

**Fig. 5:**
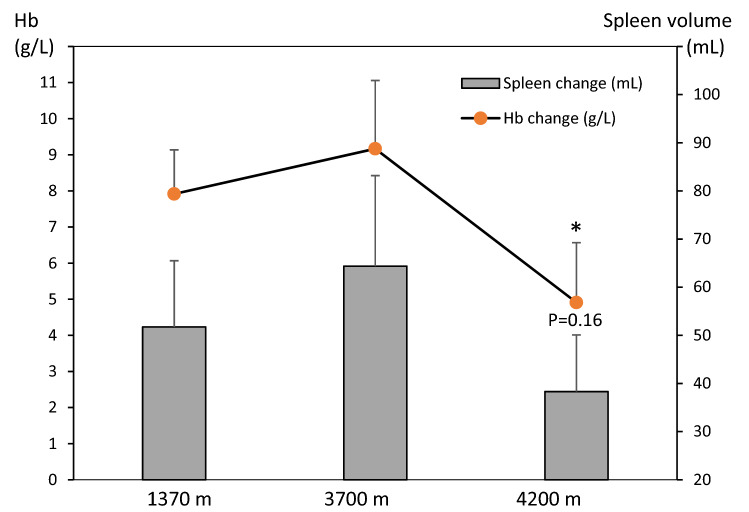
Mean (SE) change from baseline in spleen volume and hemoglobin concentration (Hb) at three altitudes; 1370 m, 3700 m and 4200 m. Values are means from 11 participants. Significant difference between Hb elevation at 3700 and 4200 m is indicated by *P < 0.05, while other differences were not significant. *P* value for spleen volume comparison between 3700 and 4200 m is shown above column

Mean (SE) baseline Hb after 10 min rest before exercise progressively increased with increasing altitude, from 138.9 (6.1) g/L at 1370 m, to 141.2 (4.1) at 3700 m (NS, power = 0.99) and 152.4 (4.0) at 4200 m (*P* < 0.05, power = 0.99 to 1370 m; *P* < 0.001, power = 0.99 to 3700 m; Fig. 4). After the step test, Hb had increased from baseline at all altitudes, to 146.8 (5.7) g/L at 1370 m (by 7.9 g/L; 5.7%; *P* < 0.001, power = 0.46), 150.4 (3.8) g/L at 3700 m (by 9.2 g/L; 6.5%; *P* < 0.01, power = 0.99) and 157.3 (3.8) g/L at 4200 m (by 4.9 g/L; 3.2%; *P* < 0.05, power = 0.96; Fig. 4). The spleen-derived Hb elevation during exercise was attenuated at 4200 m compared to 3700 m, both as absolute values (*P* < 0.05, Fig. 5). and as relative increase in Hb (*P* < 0.05, power = 0.79).

After 10 min post-exercise rest, Hb had returned to baseline at all altitudes (all NS from baseline; Fig. 4) and was 134.4 (6.7) g/L at 1370 m, 143.3 (4.3) g/L at 3700 m (NS, power = 0.99 from 1370 m) and 152.5 (4.3) g/L at 4200 m (*P* = 0.073, power = 1.00 from 1370 m; *P* < 0.05, power = 0.99 from 3700 m; Fig. 4).

### Association between spleen volume and hemoglobin concentration

Correlation analysis revealed that baseline values of spleen volume and Hb were positively associated, both as individual values (*r* = 0.442; *P* < 0.05) and for individual mean values for all altitudes (*r* = 0.667; *P* < 0.05). When altitudes were analyzed separately there was an association found at 1370 m (*r* = 0.622) and 3700 m (*r* = 0.771; both *P* < 0.05), but not at 4200 m (*r* = 0.402; NS), where spleen volume had decreased and Hb increased as a response to the increased altitude.

### Cardiorespiratory variables

**Fig. 6:**
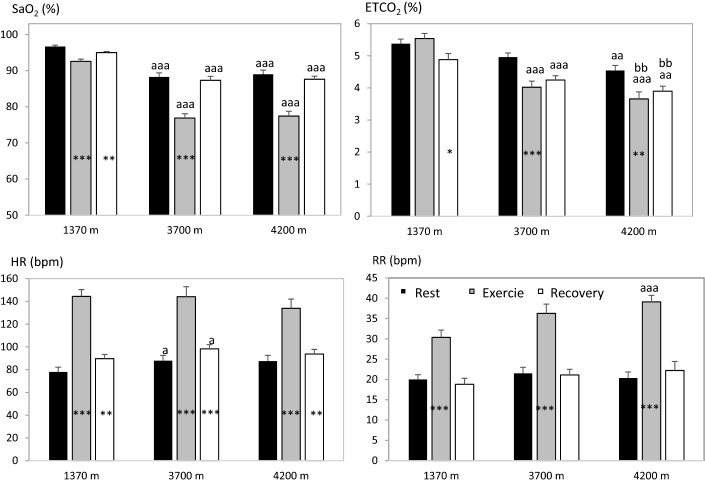
Mean (SE) values for cardiorespiratory variables before, during and 10 min after exercise at three altitudes; Kathmandu 1370 m, Beding 3700 m and Naa 4200 m. Means are based on 11 participants, except for RR, where n = 10. Arterial oxygen saturation (SaO_2_); end-tidal carbon dioxide (ETCO_2_); heart rate (HR) and respiratory rate (RR) were affected by altitude and by exercise. Significant differences between exercise and post-exercise values compared to baseline values within altitudes are indicated with *P < 0.05; **P < 0.01 and ***P < 0.001. Differences between baseline values at Kathmandu and other altitudes are indicated with a above columns for *P* < 0.05; aa for *P* < 0.01 and aaa for *P* < 0.001. There was also a difference between 3700 and 4200 m values for exercise and post-exercise ETCO_2_ indicated by bb for *P* < 0.01

Cardiorespiratory variables: HR, SaO_2_, ETCO_2_ and RR were also generally affected by the altitudes, both at rest and during exercise (Fig. 6). Baseline SaO_2_ was clearly lower at the higher altitudes, and exercise lead to a further aggravation of the hypoxia at all altitudes, an effect that was more pronounced with higher altitudes (Fig. 6a). Baseline ETCO_2_ was also lower with higher altitudes relating to the hypoxia-triggered increased respiration, as seen in the increased RR. While there was no effect of exercise on ETCO_2_ directly after exercise at 1370 m (NS), it was lowered after 10 min recovery (Fig. 6b). Exercise led to a further reduction in ETCO_2_ at the higher altitudes that remained after 10 min recovery (Fig. 6b). Baseline and recovery HR were higher at 3700 m than at 1370 m, and exercise increased HR at all altitudes (Fig. 6c). RR was increased at 4200 m compared to 1370 m, and increased as a result of exercise at all altitudes (Fig. 6d).

### Acute mountain sickness symptoms

None of the subjects were suffering from AMS during the trek. Eight subjects completed the LLQ, five male and three female. Among these subjects, the daily scores during the 6 days at 3700 and 4200 m ranged between 0 and 2 (mean 1.15). The baseline spleen volume and Hb did not correlate with LLQ scores, neither did their changes during exercise correlate with LLQ scores (NS). HR, ETCO_2_ and RR showed no correlation with the LLQ, but the SaO_2_ during the step test at 3700 m and 4200 m showed a correlation with the LLQ score (*r* = − 0.84; *P* < 0.01, power = 0.74). No correlation was identified between SaO_2_ and spleen- or Hb responses to exercise (NS).

## Discussion

This study clearly shows that the spleen acts as a dynamic red blood cell reservoir at high altitude. Recruitment of part of the reservoir occurs already during rest at high altitude, and during exercise, the spleen has a capacity to expel a supplementary volume of red blood cells into circulation, to cope with the aggravated hypoxia. It is evident from the transient elevation of Hb that spleen contraction has an effect on circulating erythrocyte volume. The close association between Hb and spleen volume supports this conclusion. This is in line with previous observations of simultaneous spleen contraction and Hb elevation during apnea (Baković et al. 2005) and during simulated altitude by eupneic hypoxia during rest (Richardson et al. 2008), but it has never been observed at high altitude.

This is the first clear evidence from a field observation that the human spleen has an important role in adjusting circulating Hb to fit the demands to varying degrees of hypoxia at high altitude. In this way, the un-acclimatized climber will have a means to cope with hypoxia much earlier than if only the Hb elevation caused by enhanced erythropoiesis would be available, which takes at least a week to develop (West 2012). The main functional effect would be to enhance the capacity for blood oxygen storage and transportation (Schagatay et al 2001), with known benefits at high altitude (West 2012).

The maximal capacity for the spleen to contract seems to be reached already during exercise at 3700 m, when the smallest volume of 166 mL is reached, and no further reduction is seen with exercise at the higher altitude 4200 m, thus this volume may be the smallest possible volume for the spleen to reach when “empty”. The largest volume observed, 273 mL, suggests that an average volume of about 100 mL of concentrated blood can be expelled from the spleen, and with a Hb about double that of the circulating blood (Laub et al., 1993; Stewart and McKenzie 2002) this would add the equivalent of the oxygen carrying capacity of an additional 200 mL of circulating blood. Based on an estimated blood volume in our group (Nadler et al. 1962), that would lead to an addition of approximately 4 g/L of circulating Hb. However, the maximum increase of Hb observed was about the double. There have been similar observations in other studies, e.g., by Richardson et al (2008) which concluded that the spleen contributed to 60% of the observed increase in hematocrit during hypoxic exposure. This suggests that other stagnant pools of erythrocytes may also be recruited, or that there was a shift in plasma volume induced, or possibly that the timing of the spleen volume and blood sampling was not synchronized with the minimum and maximum values, respectively.

The only previous observation of similar changes of this dynamic elevation of Hb with altitude was in a small study by Richardson et al. (2007) where it was discovered that the transient elevation in Hb during apnea at 1230 m was attenuated at 3840 m and eventually disappeared at 5100 m. This attenuation of the Hb response when individuals traveled to progressively higher altitudes suggests that the spleen may eventually reach its maximally contracted state during rest at high altitude, which means it cannot produce any further Hb elevation despite an additional hypoxic stimulus (Richardson et al. 2007). On the descent from altitude, the Hb responses during apnea returned, and were greater in magnitude than that on the same altitude during the ascent, suggesting that acclimatization had abolished the need of a tonic contraction during rest (Richardson et al. 2007). Thus, the tonic spleen contraction during rest on ascent may be a means to cope with the chronic hypoxia before acclimatization leads to elevated Hb and makes other protective mechanisms available. After this initial stage, only the severe hypoxia related to exercise will lead to blood-boosting for transiently increasing the oxygen carrying capacity, a response that would likely be very beneficial for fine-tuning oxygen delivery across a longer exposure to high altitude hypoxia when the oxygen transport system is under stress.

An enhancement of spleen function was found to be part of the acclimatization to hypoxia in rats (Kuwahira et al. 1999). Earlier studies on climbers found enhanced spleen contraction during exercise after long-term high-altitude exposure during an expedition summiting Mount Everest (Engan et al. 2014). In another group, trekking to less extreme altitude, it was observed that both spleen volume and contraction during exercise were enhanced on return to low altitude (Rodríguez-Zamora et al. 2015). The difference between the results in these two similar studies could possibly be caused by a general catabolism in the “Death Zone” above 8000 m, not allowing spleen enlargement. It was also recently observed that top climbers had larger spleens and spleen contraction during apnea, compared to recreational high altitude trekkers (Schagatay et al. 2020). Taken together these studies suggest that also the spleen itself may be subject to altitude acclimatization, which is in line with the current study’s observations of a significant spleen function in elevating Hb at altitude. Thus, the spleen may have two distinctive effects at high altitude: (1) to cope with the acute hypoxia in the un-acclimatized lowlander, before erythropoiesis has elevated Hb sufficiently to counteract it, and (2) a long-term effect increasing spleen volume and contractility with altitude exposure, as part of the acclimatization process, so that a larger dynamic red cell volume can be recruited by spleen contraction during exercise and stored away in the spleen during rest, to reduce blood viscosity.

Both our present study and the results cited above seem to be conflicting with a study by Sonmez et al (2007), in which it was reported that long-term exposure to high altitude in lowlanders resulted in reduced splenic volume and increased Hb across 6 months. In that time, substantial altitude acclimatization has elevated Hb via increased erythropoiesis, and it is not clear how this relates to the relatively short-term adjustments seen in the current study.

Our present results do not support the conclusions from the recent study by Purdy et al (2018) that the spleen does not mobilize erythrocytes during ascent to high altitude. In that study, baseline spleen volume was not found to differ between the altitudes 1045 m, 3440 m, and 4240 m, neither did the spleen contract as a stress response to handgrip exercise at high altitude (Purdy et al. 2018). In the same study, spleen contraction was seen at all altitudes on injection of phenylephrine hydrochloride, and the authors concluded that the spleen does not contribute to acclimatization to high-altitude hypoxia, due to alterations in spleen reactivity to increased sympathetic activation at altitude (Purdy et al. 2018). The spleen is mainly innervated by sympathetic nerves with both α- and β-adrenoceptors (Ayers et al. 1972) and spleen contraction can probably be induced by both neural input and catecholamine release (Stewart and McKenzie 2002). While resting spleen volumes observed by Purdy et al (2018) at 1045 m (273 mL), were similar to what we observed at 1370 m (252 mL), their handgrip exercise at 1045 m only caused a spleen contraction of 8%, while in our study, the step test caused a 21% spleen contraction. Likewise, the Hct in their study increased from baseline by 4%, while in our study, by 6%. This suggests that the handgrip exercise is not as powerful a stimulus as the exercise with large muscle groups causing aggravated hypoxia during step test in our study. Furthermore, in the study by Purdy et al (2018), the shrinkage of the spleen observed after phenylephrine infusion could be caused by eliciting spleen vasoconstriction, thus by a different mechanism than the physiological neural stimulation. There could also be other vascular responses from injection of the phenylephrine bolus, e.g., overstimulation of the baroreceptors, which could have blunted the endogenous sympathetic activity. Therefore, we consider the study by Purdy et al. (2018) inconclusive of whether spleen contraction occurs during exercise at high altitude. Their lack of effect of altitude on resting spleen volume remains contrasting to our observations. Purdy et al (2018) thus suggested that the constant spleen volume at different altitudes is a result of a decreased reactivity to sympathetic activity but while there may be a long-term downregulation of beta-adrenoreceptors with chronic hypoxia (Richalet et al 1988), this may not likely have developed in these relatively un-acclimatized subjects. While both the studies by Richardson et al. (2007) and Purdy et al (2018) may suggest that splenic reactivity was reduced at high altitude, the present study involving whole-body exercise induced a sufficient stressor to manifest as a powerful response also at high altitude. However, we suggest that the spleen response will likely reach a “roof” due to its anatomical properties, which appears to be the case at the highest altitudes.

The input responsible for spleen contraction in our study is most likely the hypoxia during rest and the aggravated hypoxia during whole-body exercise, in accordance with earlier findings (Richardson et al. 2008). Hypercapnia has also been found to trigger spleen contraction during apnea (Richardson et al. 2012) but would not likely be involved at high altitude due to the increased ventilation. In our study, the RR increased with increasing altitude and baseline ETCO_2_ was reduced, with further changes with exercise. Exercise in itself may have contributed as it has been found to directly induce spleen contraction (Laub et al. 1993; Stewart and McKenzie 2002).

An important role of the spleen as a dynamic red cell reservoir for predicting performance in apneic divers has been reported in several studies (Schagatay et al 2001; Bakovic et al. 2003; Schagatay et al 2012, Ilardo et al 2018) and we interpret from our current results that it could have the same role in humans exposed to high altitude. We recently reported a negative correlation between individual spleen volume during rest in Kathmandu at 1370 m and the incidence of symptoms of acute mountain sickness (AMS) in trekkers going to Mt Everest Base Camp (*P* < 0.05; Holmström et al. 2019). However, in that study, the spleen contraction during apnea at 1370 m was not significantly associated with AMS symptoms (*P* = 0.121; Holmström et al. 2019). It was however recently found that spleen baseline volume was greater in experienced mountaineers going to climb Mt Everest, than in Mt Everest Base Camp trekkers (Schagatay et al. 2020), suggesting that spleen function is important for successful climbing at high altitude.  It was also recently reported that larger spleens and more powerful contractions were present in the Sherpa population of high altitude origin, compared to Nepali lowlanders (Holmström et al. 2020). A difference between Sherpas currently residing high and low, respectively, was also observed, suggesting that not only genetic factors but also exposure to hypoxia may determine spleen size (Holmström et al. 2020). 

As an important step to understand spleen function at high altitude better, the current study shows that the spleen was more contracted during rest with higher altitudes, which was reflected by a progressively higher baseline Hb. We, therefore, speculate that spleen contraction during rest could be responsible for at least part of the transient early elevation of Hb described previously, which has been attributed to a reduced plasma concentration due either to dehydration or hormonal changes at high altitude (reviewed by West 2012). We thus interpret our result as showing an alternative explanation for the phenomenon of early elevation of Hb in newcomers to altitude, at least in strong responders. The obvious benefit of the observed mechanism would be to fine-tune circulating Hb between the oxygen demands and the aim to reduce viscosity. Thus, Hb can be optimized between—on the one hand—enhanced oxygen demands with the more severe hypoxia with exercise and—on the other—that part of the red cell supply is stored in the spleen between bouts of exercise to reduce viscosity and thereby limit the strain on the cardiovascular system related to the polycythemia. This spleen-derived regulation of circulating Hb to meet the short-term demands could possibly allow more efficient use of the limited resources during the hypoxic stress at high altitude. This original finding suggests a possible mechanism whereby a large and contractile spleen could enhance high-altitude performance which should be further studied.

We suggest that the observed transient Hb elevation with spleen contraction during work at high altitude could help explain the association between individual spleen volume and AMS symptoms, as reported by Holmström et al (2019), although with our limited LLQ data we could not support this association. The correlation between LLQ score and SaO_2_ during exercise in our study, despite a limited sample, supports that people with high SaO_2_ may likely suffer less from AMS, in accord with several other studies (West 2012).

### Study limitations

By necessity, a field study like this has restrictions on which laboratory equipment can be used, and has to rely on smaller monitors and often simpler methods than in a stationary laboratory, as all equipment has to be carried on the back to location, and the laboratory has to be rapidly mounted before tests can begin at every location. The data from the equipment in this study have, however, been compared to results obtained with more advanced monitors in stationary laboratories at high altitude (e.g., Holmström et al 2019) and been found to be in agreement.

Full measurements of minute ventilation and cardiac output would have been valuable. Measuring RR is not sufficient to determine ventilation, just as HR is not enough to determine cardiac output. However, these variables can give an indication of the effects of the regulatory influence of combined hypoxia and hypocapnia during exercise at high altitude. The ETCO_2_ was recorded in %, while measurements using mmHg would have been more useful with respect to determining effects on cerebral blood flow.

We recruited 12 subjects from a trekking group consisting of 14 lowlanders, but drop-out is common in straining field conditions. After 1 of the subjects did not complete all tests, the number of subjects was 11, which was just sufficient according to our power calculations prior to the expedition, and only 8 subjects filled out the LLQ self-assessment form. We believe that this limited sample does not allow negative conclusion on the correlation between spleen volume and physiological variables or LLQ. The small group studied is a limitation of this study.

## Conclusions

We found that the spleen contracts during rest with increased altitude and mobilizes stored red blood cells, and suggest that this could be responsible for at least part of the transient early elevation in Hb often observed during ascent to high altitude. The baseline tonic contraction during rest could give the climber a faster access to elevated Hb than the erythropoiesis-derived increase occurring during acclimatization. The response could be important as a means to cope with high-altitude hypoxia, at least in strong responders.

When not fully contracted during rest, the spleen contracts further during exercise as a response to the aggravated hypoxia, which will increase oxygen delivery to tissues to cope with transient acute hypoxia. This response apparently fine-tunes circulating Hb by optimizing the level between requirements for sufficient oxygen delivery and reduced sheer stress to the circulatory system. This response likely helps the climber to manage oxygen supplies better. We suggest that the main factor initiating spleen contraction in both rest and exercise at high altitude is the severity of the hypoxia. Based on the current study and that of Richardson et al. (2007) we concluded that the response magnitude with aggravated hypoxia depends on the prior level of contraction during rest at a given altitude, and that Hb elevation is therefore attenuated at higher altitudes. We concluded that spleen function is important at high altitude and deserves further study.

## Abbreviations

AMS: Acute mountain sickness
ETCO_2_: End-tidal carbon dioxide
Hb: Hemoglobin concentration
HR: Heart rate
RR: Respiratory rate
SaO_2_: Arterial oxygen saturation
